# Synthetic PPAR Agonist DTMB Alleviates Alzheimer’s Disease Pathology by Inhibition of Chronic Microglial Inflammation in 5xFAD Mice

**DOI:** 10.1007/s13311-022-01275-y

**Published:** 2022-08-02

**Authors:** Eunji Oh, Jeong-Hwa Kang, Kyung Won Jo, Won-Sik Shin, Young-Hun Jeong, Byunghee Kang, Tae-Young Rho, So Yeon Jeon, Jihoon Lee, Im-Sook Song, Kyong-Tai Kim

**Affiliations:** 1 Department of Life Sciences, Pohang University of Science and Technology (POSTECH), 77 Cheongam-Ro, Nam-gu, Pohang, 790-784 Republic of Korea; 2 College of Pharmacy, Dankook University, Cheonan, 31116 Republic of Korea; 3 College of Pharmacy, Kyungpook National University, Daegu, 41566 Republic of Korea

**Keywords:** Alzheimer's disease, Neuroinflammation, Microglia, Astrocyte, PPAR

## Abstract

Abnormal productions of amyloid beta (Aβ) plaque and chronic neuroinflammation are commonly observed in the brain of patients with Alzheimer’s disease, and both of which induce neuronal cell death, loss of memory, and cognitive dysfunction. However, many of the drugs targeting the production of Aβ peptides have been unsuccessful in treating Alzheimer’s disease. In this study, we identified synthetic novel peroxisome proliferator-activating receptor (PPAR) agonist, DTMB, which can ameliorate the chronic inflammation and Aβ pathological progression of Alzheimer’s disease. We discovered that DTMB attenuated the proinflammatory cytokine production of microglia by reducing the protein level of NF-κB. DTMB also improved the learning and memory defects and reduced the amount of Aβ plaque in the brain of 5xFAD mice. This reduction in Aβ pathology was attributed to the changes in gliosis and chronic inflammation level. Additionally, bulk RNA-sequencing showed that genes related to inflammation and cognitive function were changed in the hippocampus and cortex of DTMB-treated mice. Our findings demonstrate that DTMB has the potential to be a novel therapeutic agent for Alzheimer’s disease.

**Graphical abstract:**

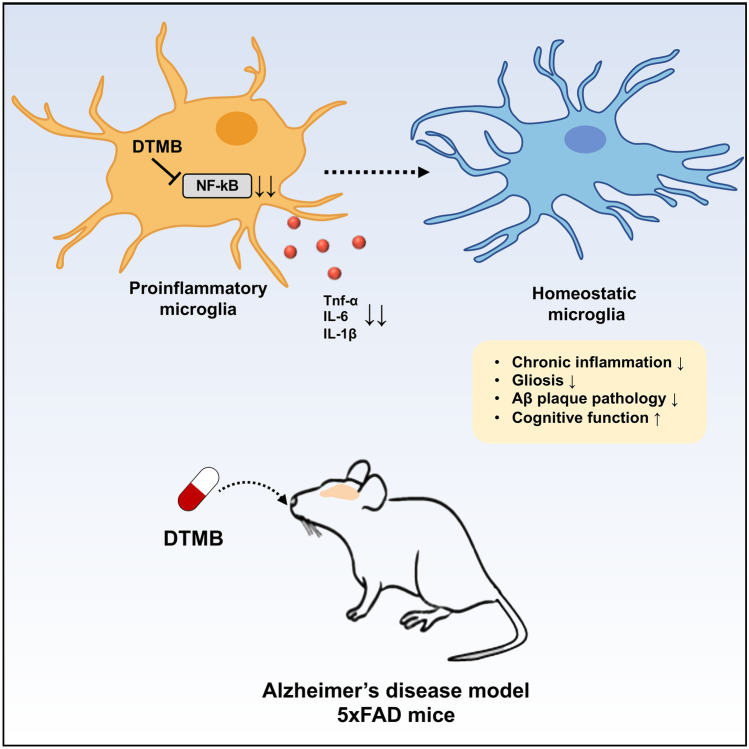

**Supplementary Information:**

The online version contains supplementary material available at 10.1007/s13311-022-01275-y.

## Introduction

Alzheimer’s disease is the most common neurodegenerative disease that leads to cognitive dysfunction, memory loss, and behavior abnormality [1]. In the brain of patients with Alzheimer’s disease, abnormal amyloid beta (Aβ) aggregates and phosphorylated tau fibrillary tangles are commonly observed as disease hallmarks [2, 3]. These protein aggregates can disrupt the function of intracellular organelles, thereby triggering neuronal cell death and synaptic failure [4]. Although there have been various attempts to develop a therapeutic agent based on Aβ peptide production mechanism, none was successful [5]. Therefore, the number of drug candidates targeting inflammation and other modes of action are increasing compared to the drug candidates that target the production of amyloid or tau aggregates [6].

Chronic inflammation is considered one of the main causes of various diseases, such as cardiovascular disease, cancer, metabolic diseases, and neurodegenerative diseases [7, 8]. The inflammatory response is a defense system that protects the body against pathogens by activating immune cells, but when inflammation becomes chronic and systemic, it increases the production of proinflammatory cytokines, leading to cell death [9, 10]. An excessive inflammatory response contributes to the pathological progression of many neurodegenerative disorders [11]. Microglia, which is a resident immune cell in the brain, eliminates unnecessary synapse or abnormal protein aggregates through phagocytosis to sustain proper synaptic circuit and maintain brain homeostasis [12, 13]. However, it has been reported that aged and overactivated microglia show a different phenotype expressing a large amount of proinflammatory cytokines, such as TNF-α, IL-6, and IL-1β, and inducing chronic inflammation in the brain [14].

Peroxisome proliferator-activating receptor (PPAR), which is a ligand-mediated transcriptional factor, chiefly regulates glucose and lipid metabolism [15]. In a previous study, PPAR was widely studied as a target for metabolic diseases such as diabetes and dyslipidemia [16]. However, in recent years, PPAR has shown its potential as a therapeutic target for neurological disorders through its function in the suppression of chronic inflammation. It has been observed that all PPAR subtypes suppress the inflammatory response in peripheral tissue by decreasing transcriptional activation by NF-κB [17]. PPARα and PPARδ negatively regulate the transcriptional expression of proinflammatory cytokines, like TNF-α and IL-6, by interrupting the interaction between NF-κB and the target gene [18, 19]. In addition, PPARγ inhibits the transcriptional role of NF-κB by restraining the nuclear translocation of NF-κB by increasing its deacetylation by Sirt1 and by increasing the proteasomal degradation of NF-κB as its E3 ligase [20, 21].

In the current study, we aimed to discover a novel synthetic PPAR agonist that improves chronic inflammation in the Alzheimer’s disease. We investigated the anti-inflammatory effect of DTMB on microglia in the pathological condition similar to chronic inflammation during Alzheimer’s disease through the treatment of human aggregated Aβ and LPS. In addition, DTMB treatment alleviated memory dysfunction and progression of pathology in the brain of 5xFAD mice model. RNA-sequencing data and gene expression pattern in isolated adult microglia support the therapeutic potential of DTMB as a novel drug for Alzheimer’s disease.

## Materials and Methods

### PPAR Binding Luciferase Assay

HEK293A cells were transfected with the GAL4-reporter plasmids and pGL4.74 (Promega, Madison, WI, USA) as an internal standard through electroporation using a Neon Transfection System (Invitrogen, Carlsbad, CA, USA). All vector materials were provided by Professor Yuichiro Kanno at Toho University. Cells were treated with DTMB and each positive control compound for 24 h after overnight incubation. Cells were then harvested and resuspended in luciferase lysis buffer (Promega), followed by incubation on ice for 10 min. Cell debris was removed by centrifugation at 20,000 × *g* at 4 °C for 10 min, and the supernatants were used for luciferase assay. Using a dual-luciferase reporter assay system (Promega), luciferase activities were measured according to the manufacturer’s instructions. Experimental firefly luciferase activities were normalized against those of Renilla luciferase.

### Cell Viability Assay (MTT Assay)

Cell viability was measured by chromogenic assay involving the biological reduction of 3-(4,5-dimethyl-thiazol-2-yl)-2,5-diphenyltetrazoliumbromide (MTT) to formazan in living cells. Briefly, cells were seeded in 96-well plates in a final volume of 100 μL. After 24 h, the culture media were replaced with fresh media containing DTMB compounds, and the cells were cultured for another 48 h. Next, MTT (5 mg/mL in PBS) was added to the culture medium. The cells were incubated for 1 h in the dark at 37 °C, and the supernatant was then removed from the well. Total MTT solvent (4 mM HCl and 0.1% Nonidet P-40, both in isopropanol) was added to dissolve the formazan crystal, and absorbance was measured at 570 nm using a microplate reader Infinite 200 Pro NanoQuant microplate reader (TECAN, Switzerland).

### Molecular Docking

Crystal structures of ligand binding domain of human PPAR family (PPARα: 4BCR, 3VI8, 5HYK; PPARδ: 5U3Q, 5U46; PPARγ: 3U9Q, 5YCP, 5JI0) were prepared from RCSB Protein Data Bank. The energy of DTMB or other agonists of PPARs (WY14643, GW501516, and rosiglitazone) were minimized using open babel in PyRx software for further docking analysis [22]. Using AutoDock Vina in PyRx software, molecular docking and calculation of predicted binding energy was performed to find docking position of ligands to PPARs [22, 23]. The grid map for docking was covered on overall structures of the macromolecules. Docking results were visualized using PyMol visualization system. Hydrogen bonds were predicted by PyMol software.

### CNBr-Bead Conjugation Assay

CNBr Sepharose 4B beads were prepared and activated according to the instruction. 100 mg of CNBr activated Sepharose 4B beads were incubated with drugs or DMSO for 18 h at 4 °C. The drug conjugated beads reacted to his-tagged recombinant PPAR α, β/δ, and γ protein. After 24 h of incubation, we confirmed whether recombinant protein binds to the drug conjugated bead through western blotting experiment.

### Pharmacokinetics and Brain Distribution of DTMB

To investigate the pharmacokinetics and brain distribution of DTMB, fifty-six male C57BL/6 mice (22–26 g) were fasted with water ad libitum for 16 h before oral administration of DTMB and randomly divided into seven groups (*n*=8 per each sampling time point). Mice were administered with a DTMB suspension in 1% carboxymethylcellulose at a dose of 50 mg/kg via oral gavage, and blood samples (approximately 0.1 mL) were collected at 0, 0.16, 0.5, 1, 4, 8, and 24 h via the abdominal artery. Subsequently, whole brain tissues were isolated, weighed, and homogenized with two volume of ice-cold 80% methanol solution using a tissue grinder. Blood samples were centrifuged at 12,000 g for 1 min to separate plasma. Aliquots of plasma (30 µL) and brain homogenate (50 µL) samples were stored at − 80 °C until analysis. Concentrations of DTMB in plasma and brain homogenate samples were analyzed using an Agilent 6470 triple quadrupole liquid chromatography-mass spectrometry (LC–MS/MS) system (Agilent, Wilmington, DE, USA). Aliquots of plasma (30 µL each) and brain homogenate (50 µL each) were added to 100 μL of internal standard solution (IS; berberin 0.1 ng/mL in methanol) and vigorously mixed for 5 min. After centrifugation at 16,000 g for 10 min, a 5 µL aliquot of the supernatant was injected into the LC–MS/MS system. DTMB was separated on a Synergi Polar RP column (2.0 × 150 mm, 4 μm particle size; Phenomenex, Torrence, CA, USA) using an isocratic mobile phase consisting of 10 mM ammonium formate buffer (10%) and methanol (90%) containing 0.1% formic acid at a flow rate of 0.25 mL/min. Quantification of the analyte peaks was carried out at m/z 513.2 → 283.0 for DTMB (TR (retention time) 4.7 min) and m/z 336.1 → 320.0 for berberine (TR 2.9 min) in a negative ionization mode with optimized fragmentor of 135 V and collision energy of 25 or 30 eV, respectively. The pharmacokinetic parameters of DTMB in the plasma and brain were determined by the non-compartmental analysis (WinNonlin® 5.1; Pharsight, Mountain View, CA, USA). Elimination half-life (*T*_1/2_) was calculated from the elimination coefficient (*K*) of DTMB concentrations. Cmax indicated the maximum plasma concentrations and Tmax indicated time to reach Cmax. AUClast and AUCinf were calculated from the area under plasma concentrations curve from zero to the last time point and to infinity, respectively.

### Cell Culture

The BV2 mouse microglia cell line was received from Professor K. H. Suk (Kyungpook National University, Korea). BV2 cells were cultured with Dulbecco’s Modified Eagle Medium (DMEM) (Hyclone, Logan, UT, USA), containing 5% fetal bovine serum (FBS, Hyclone) and 50 μg/mL of gentamicin (Lonza, Switzerland), in a 5% CO_2_ incubator with a humidified atmosphere at 37 °C. RAW 264.7 macrophage cells were cultured with DMEM that contains 10% FBS and 1% penicillin/streptomycin in a 5% CO_2_ incubator with a humidified atmosphere at 37 °C.

### Primary Microglia Culture

Primary microglia culture was conducted as described in a previous study [24]. Mice at postnatal day 3 mice were used for the preparation of primary microglia. Newborn mice pups were anesthetized in ice and then sacrificed. Isolated cortex and hippocampus were collected after removing the meninges. Brain tissues were dissociated by incubating in HBSS (Gibco, Gaithersburg, MD, USA) with trypsin (2.5%) for 15 min. Cell homogenates were then incubated in M-CSF (20 ng/mL, R&D Systems) supplemented with DMEM-F12 medium with 10% fetal bovine serum, 10% horse serum, 1% GlutaMAX (Gibco, MD, USA), and 1% penicillin/streptomycin (Welgene, Korea). After 10 days, microglia were purified through shaking at 260 rpm for 2 h.

### Griess Assay

Griess assay (Promega) was performed under manufacturer’s instruction to measure the concentration of nitrite after inducing the inflammatory response in RAW 264.7 macrophage cells by LPS treatment. The well-known PPAR selective agonists, such as WY14643 (PPARα), GW501516 (PPAR-β/δ), and rosiglitazone (PPARγ), were used as positive controls.

### ELISA

We measured cytokine level (TNF-α, IL-1β, and IL-6) in the media of LPS (1 μg/mL) and human Aβ_1-42_ (5 μg/mL)-treated BV2 microglia cells and primary microglia. Human Aβ_1-42_ (AnaSpec, Fremont, CA, USA) were pre-aggregated in the incubator for 24 h before the treatment to the cells. BV2 cells and primary microglia cells were seeded on 12-well plates (SPL) at a density of 1 × 10^5^ cells/well cultured in 1 mL of complete media. After 12 h, LPS or human Aβ_1-42_ were treated to cells and were further incubated for 24 h with DTMB. ELISA experiments were conducted by using mouse TNF-α ELISA kit (Invitrogen, BMS607-3TEN), mouse IL-1β ELISA kit (Invitrogen, BMS6002), and mouse IL-6 ELISA kit (Invitrogen, BMS603-2) according to the manufacturer’s instruction.

### Animals

5xFAD (Tg6799) mice were purchased from the Jackson Laboratory (Bar Harbor, ME, USA) and male transgenic mice were bred with B6/SJL hybrid female mice. Genotyping was conducted by PCR analysis of tail cut samples. Eight-week-old female mice were used for administration of drug in this study and wild-type littermates were used as controls. All procedures related to animal behavioral experiments were approved by Pohang University of Science and Technology Institutional Animal Care and Use Committee (POSTECH IACUC). All animal experiments were carried out according to the provisions of the Animal Welfare Act, PHS Animal Welfare Policy, and the principles of the NIH Guide for the Care and Use of Laboratory Animals. All mice were maintained under conventional conditions at the POSTECH animal facility under institutional guidelines. For the pharmacokinetic and brain distribution study, C57BL/6 mice (8-week-old, male) were purchased from Samtako Co. (Osan, Kyunggi-do, Korea). Animals were acclimatized for 1 week in an animal facility at Kyungpook National University. All procedures were approved by the Animal Care and Use Committee of Kyungpook National University (KNU IACUC protocol code 2022–0126).

### Drug Treatment

DTMB was synthesized from Daejung Chemicals (Korea) and was dissolved in dimethyl sulfoxide (Sigma Aldrich, St. Louis, MO, USA) for cell-based experiments. To evaluate the effects of DTMB on 5xFAD mice, DTMB (10 mg/kg) and vehicle (1% carboxymethylcellulose and 10% DMSO) were orally administrated to four groups (wild-type, wild-type + DTMB, 5xFAD, and 5xFAD + DTMB) with a 6 day per week for 3 months.

### Y-Maze

The Y-maze test was conducted using a black y-shaped maze apparatus that has three equal-sized arms (40 × 3 × 12 cm) separated at 120° angles. The mice were placed into the same side of the arm at every trial and freely moved for 8 min under the dim light conditions (50 lx). To measure spatial memory, the number of arm entries to other sides was measured. The percentage of spontaneous alternation was calculated using the following formula: $100\times (\frac{Actual\; Alteration}{(Total\; \#\; of\; arm\; entrance-2)})$. Sample sizes were 20–25 mice per group.

### Morris Water Maze

A white water maze tank (120 cm diameter) was filled with water containing white non-toxic tempera paint to make the water opaque. The maze tank was divided into four quadrants, and four different visual cues were placed at each cardinal point. The water temperature was set to 20–22 °C. The Morris water maze experiment was divided into a training session, during which the mice were placed in the tank to find the platform for five consecutive days, followed by a probe test on day 6. Each mouse was trained over three trials per day and the starting quadrant was different for each trial. The duration of each training trials was 1 min and we guided the mice to the platform thereafter. In the probe test on day 6, the platform was removed, and each mouse was allowed to swim for 60 s. Target quadrant occupancy was measured automatically using a SMART v2.5 video tracking system (Panlab, TX, USA). Sample sizes were 20–25 mice per group.

### Immunohistochemistry

Mice were perfused with ice-cold phosphate-buffered saline (PBS). Brain tissues were separately used for either molecular analysis or immunohistochemistry. The right hemisphere was fixed with 4% PFA and was transferred to a sucrose solution (30%) for 24 h. The right hemisphere was embedded and frozen in optimal cutting temperature compound on dry ice. Coronal Sections (18 μm thick) that included the hippocampus were cut and prepared on slide glasses. Tissue sections were then permeabilized and blocked using a blocking solution (5% goat serum and 0.2% Triton X-100 in PBS) for 1 h at room temperature. Primary antibody incubation was performed overnight at 4 °C using an anti-6e10 antibody (Biolegend, Japan, 1:100), anti-Iba1 antibody (Wako, Japan, 1:200), and anti-GFAP antibody (Abcam, Cambridge, UK, 1:200). After the washes, sections were incubated with the appropriate secondary antibody (1:500) for 1 h at room temperature. Slides were counterstained in Hoechst and then mounted in fluorescence mounting medium (Dako, Denmark). All images were taken through an Axioplan2 microscope (Zeiss, Germany). Fluorescence was measured by calculating % area and was analyzed using ImageJ. Sample sizes were 10–15 mice per group.

### Soluble and Insoluble Aβ Extraction

To investigate soluble and insoluble Aβ components, two extraction steps were performed. Left hemisphere samples were homogenized in cold Tris-buffered saline (TBS consisting of 50 mM Tris-pH 7.5 and 150 mM NaCl) with a protease inhibitor cocktail (Thermo Fisher Scientific, Waltham, MO, USA) at a concentration of 100 mg/mL. After centrifugation at 15,000 rpm for 1 h at 4 °C, the supernatant of the samples was used to conduct western blotting. Insoluble protein was extracted from the pellet by dissolving in 5 M guanidine buffer (5 M guanidine HCl and 150 mM NaCl-pH 7.5). Soluble and insoluble protein samples were stored at −80 °C.

### Western Blot Analysis

Cells were lysed with lysis buffer containing 50 mM Tris (pH 7.4), 140 mM NaCl, 5 mM EDTA, and a protease inhibitor tablet, followed by sonication. Lysate protein concentrations were determined using Bradford reagents (AMERSCO, MA, USA). Proteins were resolved by sodium dodecyl sulfate (SDS) polyacrylamide gel electrophoresis, transferred to nitrocellulose membranes (Pall Corporation, New York, NY, USA), and incubated with blocking buffer (5% nonfat dry milk in TBS and 0.1% Tween 20) for 30 min. Western blot analyses were performed using primary antibodies against NF-κB, NLRP3, ASC, nicastrin, pen2 (Cell Signaling Technology, Danvers, MA, USA), Aph1 (Invitrogen), amyloid C-terminal fragment (Santa Cruz Biotechnology, Dallas, TX, USA), and GAPDH (Bethyl Laboratories, Mongomery, TX, USA). Secondary antibodies of rabbit (Promega), rat, and goat (Bethyl Laboratories) were used, and detection was performed with SUPEX ECL reagent (Neuronex, Korea) and an ImageQuant LAS-4000 (GE Healthcare, MA, USA), according to the manufacturer’s instructions. The integrated blot density was quantified through ImageJ.

### Filter-Trap Assay

The insoluble fraction of protein lysates in 5xFAD mice was diluted in 1% SDS-PBS and boiled at 95 °C for 5 min. The membrane was pre-equilibrated with 1 × TBS. A dot blotter apparatus (Bio-Rad Laboratories, Hercules, CA, USA) was used for sample application, and 6e10 antibody was used to detect Aβ contents from brain lysates of 5xFAD mice treated with DTMB. Sample sizes were 3 mice per group.

### Reverse Transcription (RT-PCR) and Real-Time Quantitative PCR (qPCR)

Total RNA was isolated using TRI Reagent (Molecular Research Center, Cincinnati, OH, USA). RNA was reverse transcribed using the ImProm-II™ Reverse Transcription System (Promega) according to the manufacturer’s instructions. For detection and quantification, a StepOnePlus Real-Time PCR System (Applied Biosystems, Foster City, CA, USA) was used with FastStart Universal SYBR Green Master (Roche, Basel, Switzerland). Real-time qPCR data were analyzed using the comparative C_T_ method. Sample sizes were 10–15 mice per group.

### Magnetic Sorting of Adult Microglia

To isolate the adult microglia from DTMB- or vehicle-treated 5xFAD mice brain, magnetic-activated cell sorting (MACs) system was used. Whole brain tissue was dissociated to a single cell level using gentleMACs dissociation kit (Miltenyi Biotech, Bergisch Gladbach, Germany) according to manufacturer’s instruction. To separate the immune cell fraction, 47% percoll gradient was applied to single cell mixture and centrifuged at 2,000 rpm for 10 min. Adult microglia was isolated by LS column after incubating with anti-CD11b MicroBeads (Miltenyi Biotech). Separated adult microglia were used to analyze the expression of genes related to microglia activation and inflammation by performing RT-PCR. Sample sizes were 3 mice per group.

### RNA Library Preparation and Data Analysis

RNA-seq libraries were prepared using the NEBNext® Ultra II Directional RNA Library Prep Kit for Illumina (BioLabs, MA, USA) and sequenced on Illumina HiSeq 2500 (San Diego, CA, USA). RNA-seq reads with adapter sequence were trimmed using cutadapt [25] and aligned to the Mouse Ensemble Archive Release 100 by STAR [26]. Gene expression levels were quantified by RSEM [27]. Differential expression analysis was performed by DESeq2 [28], and functional enrichment of Gene ontology (GO) terms and pathways was estimated by Metascape [29]. Pathway enrichment was estimated by GSEA [30] with the following options: “-metric log2_Ratio_of_Classes -permute gene_set.”

### Statistical Analysis

All statistical analyses were performed using GraphPad Prism version 9.2. Normality test was performed before the statistical analysis using GraphPad system. Comparisons between two groups were analyzed by two-tailed unpaired Student’s *t* tests. For comparisons between more than two groups, a one-way or two-way analysis of variance (ANOVA) was used with Tukey’s test. A *p*-value less than 0.05 was considered statistically significant. All quantitative data are presented as the mean ± standard error of the mean (SEM) except pharmacokinetic data which are presented as the mean ± standard deviation (SD).

## Results

### Identification of the Novel Pan-PPAR Agonist

**Fig. 1:**
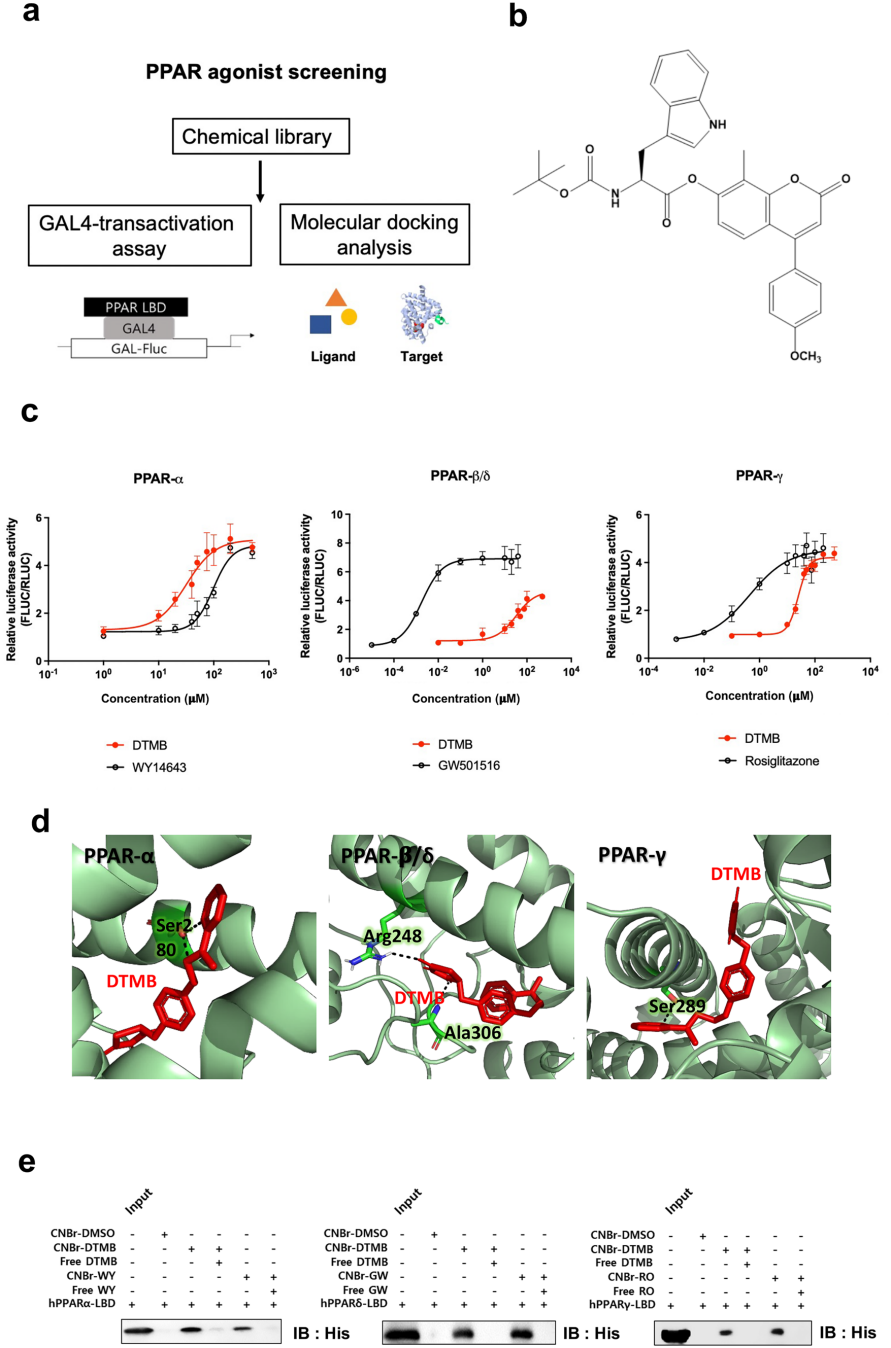
Identification of DTMB as a PPAR α/δ/γ agonist. **a** Schematic flowchart of the overall strategy for PPAR agonist screening. **b** Chemical structure of DTMB. **c** Gal4-transactivation assay. pcDNA5-GAL4-PPAR LBD, pG5-luc, and Rluc vector (as control) were transfected into HEK293A cells, which were then treated with various concentrations of DTMB for 24 h, followed by measurement of relative luciferase activity using dual-luciferase system. Cells were treated with each known PPAR ligands as positive control to compare with the response elicited by DTMB. Data are the mean ± standard error of mean (SEM, *n* = 6). **d** Molecular docking analysis. Docking positions and binding energy of DTMB to PPAR LBD (PPARα: 4BCR, 3VI8, 5HYK; PPARδ: 5U3Q, 5U46; PPARγ: 3U9Q, 5YCP, 5JI0) were predicted using PyRx and PyMol software. DTMB was predicted to have hydrogen bond to Ser280 of PPARα LBD, Arg248 and Ala306 of PPARδ, and Ser289 of PPARγ. **e** CNBr-bead pull down assay. Recombinant human PPAR LBD protein directly binds to the DTMB conjugated CNBr-bead

To identify a novel PPAR agonist, two different analyses were performed (Fig. 1a). First, a cell-based Gal4-transactivation assay was conducted to screen chemical with potential as ligand. We used a reporter construct pcDNA5/GAL4-reporter construct containing the PPAR ligand domains (α/β/γ) and measured the relative luciferase activity following the treatment with each compound from chemical library for 24 h after transfection. At the same time, molecular docking analysis was additionally performed through AutoDock Vina in PyRx software and PyMol program to increase the reliability of the drug screening. As a result, DTMB, which is a synthetic tryptophan derivative (Fig. 1b), was identified as a novel ligand capable of binding to all PPAR subtypes. Furthermore, DTMB increased luciferase activity mediated by each PPAR subtype in a concentration-dependent manner, indicating that DTMB has potential as a pan-agonist of PPAR (Fig. 1c). Comparison between the DTMB-induced luciferase activity of PPAR subtypes to each respective positive control showed that the binding affinity of DTMB was stronger than the positive control of PPARα (WY14643) and lower than the positive controls of the other PPAR subtypes (PPARδ and PPARγ) (Fig. 1c). As with the result of luciferase assay, molecular docking analysis predicted that the binding energy between DTMB and PPARα ligand binding domain (LBD) was higher than WY14643 (Fig. 1d and Table 1). To affirm the depicted binding between DTMB and PPAR, a pull-down assay was conducted using DTMB conjugated CNBr beads with the  recombinant protein of human PPARα/β/γ LBD. Western blot clearly showed that DTMB binds to three types of PPAR LBD, showing particularly stronger binding to PPARα LBD than WY14643 (Fig. 1e). These results were consistent to luciferase reporter assay and molecular docking analysis. It implicates that DTMB is a potent modulating ligand to all types of PPAR receptor.

**Table 1:** Predicted binding energy of PPAR α/δ/γ and DTMB

Target LBD model of PPARα	Predicted binding energy (ΔG) with WY14643 (kJ mol^−1^)	Predicted binding energy (ΔG) with DTMB (kJ mol^−1^)
3vi8	−7.8	−8.3
5hyk	−6.9	−8.4
4bcr_A	−7.6	−8.3
4bcr_B	−7.6	−8.6
Target LBD model of PPARδ	Predicted binding energy (ΔG) with GW501516 (kJ mol^−1^)	Predicted binding energy (ΔG) with DTMB (kJ mol^−1^)
5u3q_A	−11.4	−9.4
5u46_A	−10.9	−7.7
5u46_B	−10.7	−9.3
Target LBD model of PPARγ	Predicted binding energy (ΔG) with rosiglitazone (kJ mol^−1^)	Predicted binding energy (ΔG) with DTMB (kJ mol^−1^)
3u9q	−10.7	−7.7
5ycp	−9.9	−8.2
5ji0	−10.7	−8.0

### Pharmacokinetics and Brain Distribution of DTMB

**Fig. 2:**
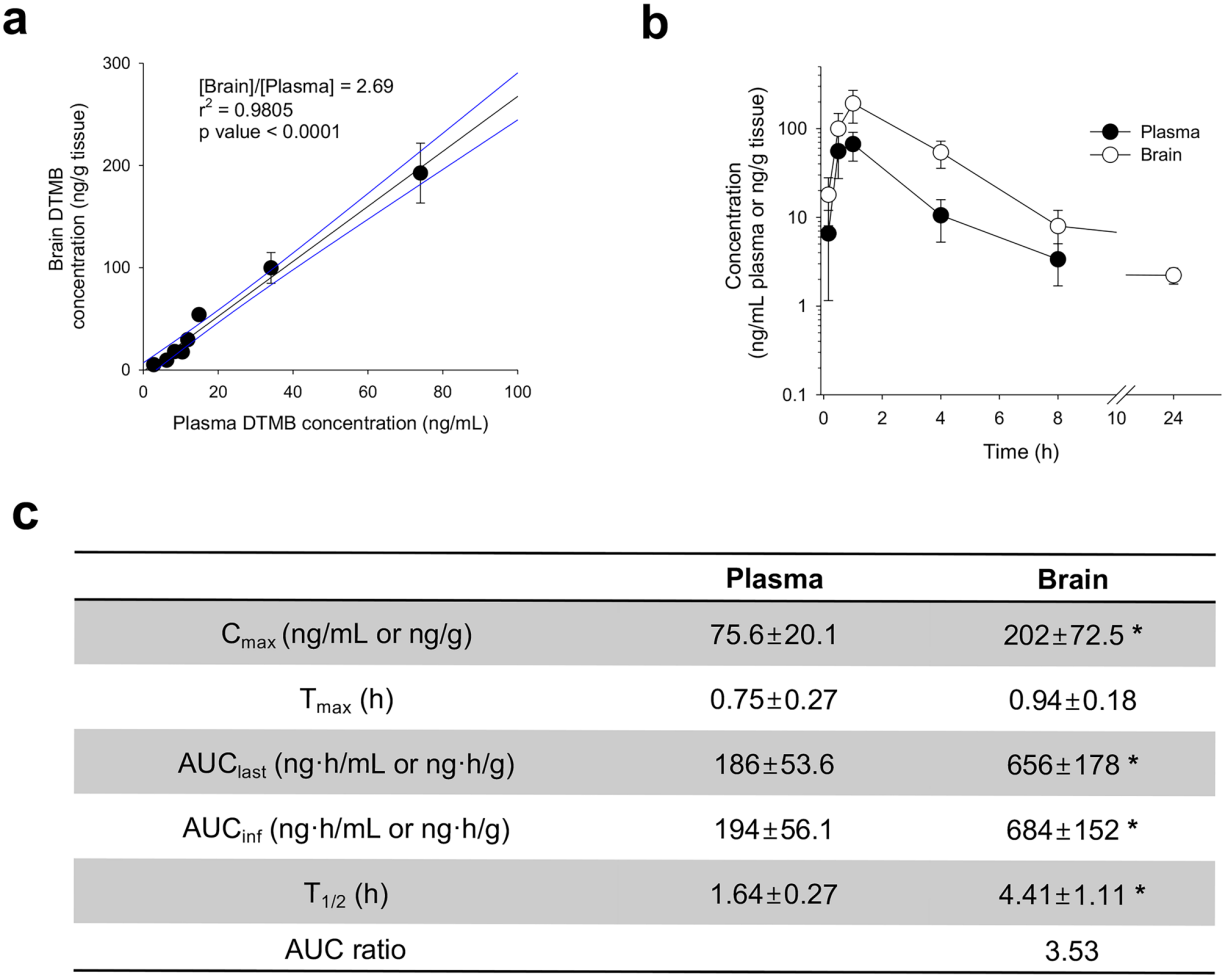
Pharmacokinetics and brain distribution of DTMB. **a** Correlations between brain DTMB concentrations and plasma DTMB concentrations after oral administration of DTMB in mice. Lines were generated from linear regression analysis and 90% confidence intervals around the geometric mean value. *r*^2^ represents the correlation coefficient and *p* represents the statistical significance for the regression analysis. **b** Plasma and brain concentration vs. time profiles of DTMB after single oral administration of DTMB (50 mg/kg) in mice. **c** Pharmacokinetic parameters of DTMB in mice after oral administrations of DTMB. Data represent mean ± standard deviation (*n* = 8 per each data point). *p < 0.05 by *t* test

For the therapeutical effect of DTMB in vivo system, we conducted to analysis of pharmacokinetics and brain distribution of DTMB. Since the brain distribution of DTMB is important for the therapeutic efficacy of Alzheimer’s disease, we measured the DTMB concentration ratio of brain to plasma in various concentrations of plasma concentration. The DTMB concentrations in the plasma and brain homogenate samples were analyzed using LC–MS/MS system. The calibration standards of DTMB in the plasma and brain homogenates were linear in the range of 0.5–500 ng/mL and 0.5–100 ng/mL, respectively, and a coefficient of determination (*r*^2^) of over 0.99 (Supplementary data 1a, b). Supplementary data 1c, d show the representative chromatograms of double blank, zero blank, calibration standard of DTMB (0.5 ng/mL of DTMB), and plasma or brain sample at 0.5 h after per oral administration of DTMB. The results revealed no significant interference peaks in the retention times of the analytes and the feasibility of our analytical method. To investigate the brain distribution of DTMB, the concentration ratio of brain to plasma was analyzed in various concentrations of plasma. The regression analysis of brain DTMB concentration over the plasma DTMB concentration revealed the linearity over the plasma concentration range of 0.63–66.9 ng/mL and the mean brain DTMB concentration ratio over the plasma DTMB was 2.69, suggesting that DTMB readily penetrates the blood–brain barrier and distributed to the brain in various plasma concentrations of DTMB (Fig. 2a). The results were consistent with the higher brain DTMB concentration compared with the plasma DTMB concentration (Fig. 2b). To compare the pharmacokinetic features of DTMB, pharmacokinetic parameters of DTMB in the plasma and brain are shown in Fig. 2c. DTMB concentration and area under concentration curve (AUC) values in the brain were significantly greater than those in plasma with significantly higher elimination half-life (*T*_1/2_). The results suggested the higher brain distribution of DTMB and prolonged maintenance of DTMB in the brain compared with plasma DTMB.

### DTMB Decreases the Inflammatory Response in Microglia Under Pathological Conditions

**Fig. 3:**
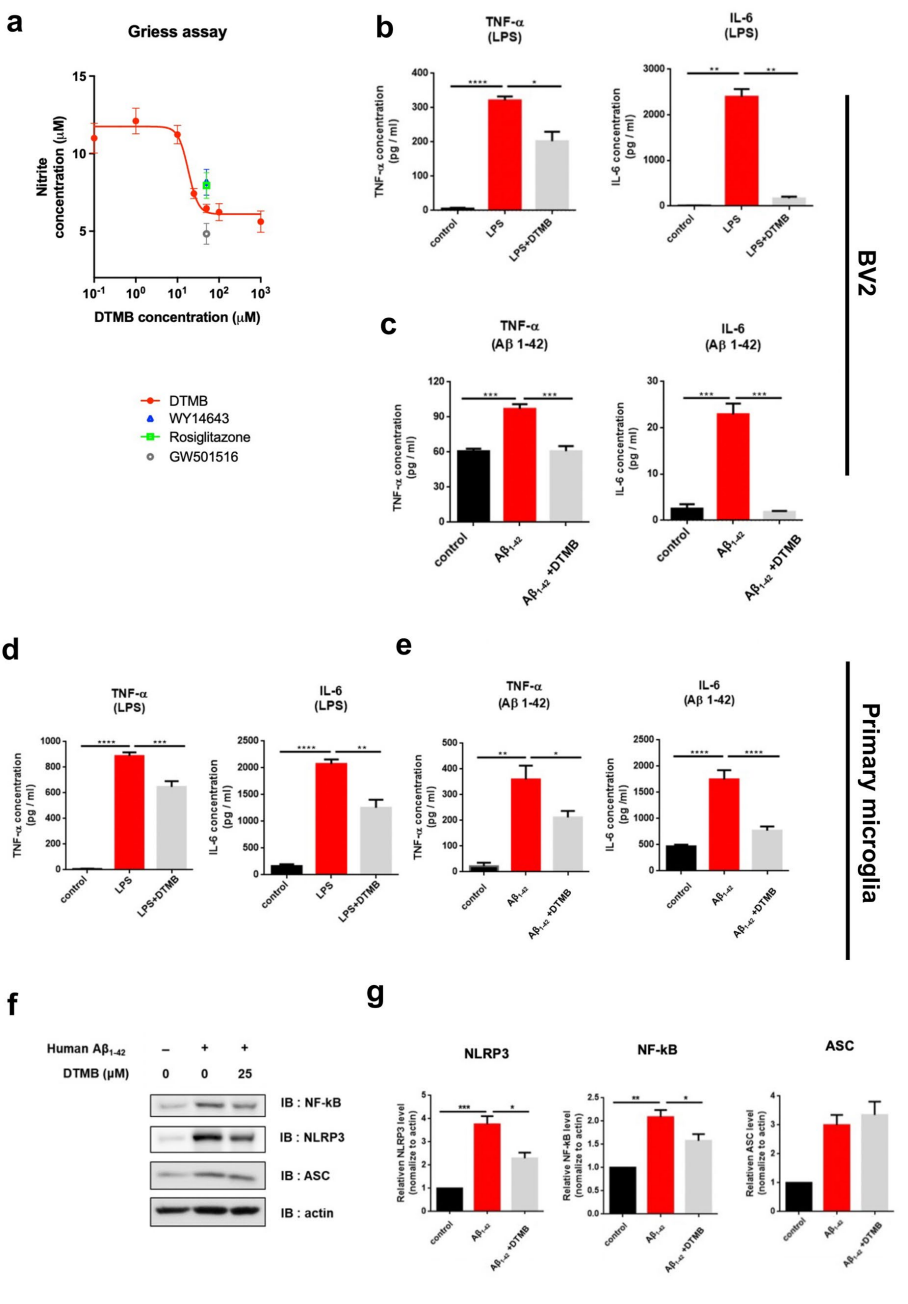
DTMB has an anti-inflammatory effect on microglia by reducing NF-κB protein level. **a** Griess assay data. RAW 264.7 macrophages were treated with LPS (1 µg/mL) to induce NO production. Cells were treated with the indicated drug for 24 h, and nitrite concentration was measured using a Griess reaction kit (*n* = 6). **b**, **c** Anti-inflammatory effect of DTMB on LPS-treated BV2 cells or primary microglia. LPS (0.5 µg/mL) was used to induce proinflammatory cytokines either with DMSO (0.1%) or DTMB for 24 h. **d**, **e** Anti-inflammatory effect of DTMB on pre-aggregated Aβ-treated BV2 cells or primary microglia. Human Aβ_1-42_ were pre-aggregated for 24 h in an incubation chamber. Aggregated Aβ (4 µg/mL) was then used to induce inflammation in cells treated with either DMSO (0.1%) or DTMB for 24 h, and the production of proinflammatory cytokines was detected. **f**, **g** Representative western blot data of NF-κB, NLRP3, and ASC from primary microglia treated with either DMSO (0.1%) or DTMB (25 µM) for 24 h. Quantification of blot intensity was determined using ImageJ. Data represent the mean ± standard error of the mean (SEM) of three independent experiments (*p < 0.05; **p < 0.01; ***p < 0.001, by one-way ANOVA)

PPAR exerts an anti-inflammatory effect by regulating the NF-κB signaling pathways [18–21]. NF-κB is an important transcriptional factor of the immune response that regulates the activation of immune cells contributing to the pathogenic processes during disease-related conditions [31]. Activation of NF-κB signaling increases the expression of proinflammatory cytokines (IL-6 and IL-1β), an inducible enzyme (iNOS), and several inflammasome factors, including members of NLRP family, ASC, and procaspase-1 [32]. Before confirming the anti-inflammatory effect of DTMB in cells, a cytotoxicity was confirmed by conducting the MTT assay. When we treated DTMB to immune cells like RAW 264.7, BV2, and HMO6 cell line, there are no cytotoxicity in a higher concentration (Supplementary data 2a). Next, we measured the production of nitric oxide (NO), which is a final product of the inflammation response, through Griess assay to evaluate the anti-inflammatory effect of DTMB on an LPS-induced inflammatory response in RAW 264.7 cells. Pretreatment of DTMB reduced NO production in a concentration-dependent manner (Fig. 3a). The treatment of cells with DTMB inhibited NO production greater than treatment with the known agonists of PPARα and PPARγ (WY14643 and rosiglitazone, respectively), but an agonist of PPARδ (GW501516) was a better inhibitor than DTMB.

To investigate the exact anti-inflammatory effect of DTMB, we measured the level of proinflammatory cytokines from BV2 microglia cells and primary cultured microglia, which were treated with either LPS or pre-aggregated human Aβ_1-42_. The level of cytokines induced by inflammatory signals (LPS or Aβ_1-42_) was dramatically reduced by DTMB treatment (Fig. 3b–e). In the primary microglia, expression of the NF-κB target genes related to chronic inflammation, namely *Il-6*, *Il-1β*, and *Inos*, was decreased. By contrast, the anti-inflammatory marker (*Arg-1*) was induced by DTMB treatment (Supplementary data 2b). Consistent with the proinflammatory cytokine level and mRNA expression, the protein levels of NF-κB and NLRP3, which are components of inflammasomes, were also decreased by DTMB. However, the protein levels of ASC were not changed in either of BV2 cells or the primary microglia cells (Fig. 2F, G and Supplementary data 1c, d). Furthermore, these responses were mediated by proteasomal degradation and not by transcriptional inhibition because there was no change in mRNA levels of *NFκB* (Supplementary data 2e, f). These results support that DTMB prominently decreases the production of proinflammatory cytokines in microglia under pathological conditions by reducing NF-κB protein level.

### DTMB Ameliorates Learning and Memory Deficits in 5xFAD Mice

**Fig. 4:**
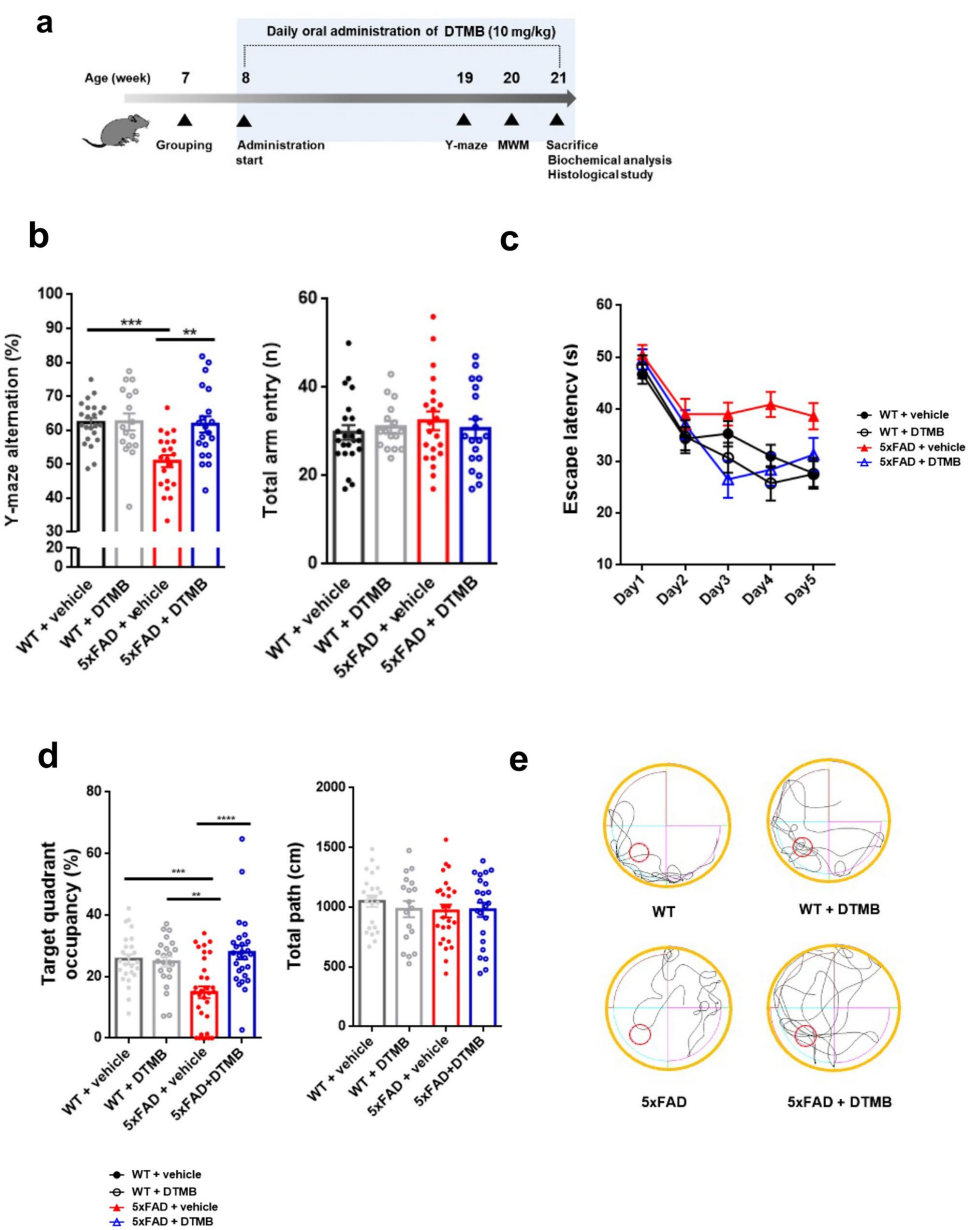
DTMB improves spatial memory and learning in 5xFAD mice. **a** Timeline of the in vivo experiment. Oral drug administration was started at the 8th week and continued for 3 months. Y-maze test and Morris water maze test were conducted to evaluate learning and memory of 5xFAD mouse. At the end of testing, all mouse brains were prepared for further biochemical and histological analysis. **b** Y-maze test. The percentage of Y-maze alternation decreased to almost 50% in transgenic mice. There were no changes of total path. **c**–**e** Morris water maze test. **c** Training process for the Morris water maze. Each mouse went through three trials of training per day to find the platform in the target quadrant. The probe test was conducted on day 6. **d**, **e** Target quadrant occupancy (%) and mouse movement were measured automatically using a Smart v2.5 video tracking system. There was no change of total path. All data are the mean ± standard error of the mean (SEM) (*n* = 25 mice per group). **p < 0.01, ***p < 0.001 by two-way ANOVA

Microglial activation is commonly observed in the brain of patients with Alzheimer’s disease and 5xFAD mice. Many studies have shown that eliminating activated microglia or treatment with anti-inflammatory drugs improves the memory defects found in Alzheimer’s disease model mice [33, 34]. In the above results, we demonstrated that DTMB has an anti-inflammatory effect on microglia after the induction of an inflammatory response through pre-aggregated Aβ. To investigate the effects of DTMB on memory function, DTMB (10 mg/kg) was orally administrated to an 8-week-old 5xFAD mice for 3 months. Behavior tests were performed after administration to examine spatial learning and memory. Further, prepared tissues were analyzed by conducting immunohistochemistry and biochemical experiment (Fig. 4a). When we treated DTMB for 3 months, there are no changes to body or tissue weight between vehicle- and DTMB-treated mice (Supplementary data 3). Through the Y-maze experiment, the spontaneous spatial memory of a mouse can be measured by the percentage of alternation. We found that the alternation of the 5xFAD transgenic mouse group decreased to nearly 50%, whereas the alternation of the DTMB-treated 5xFAD mouse group showed an improvement with a percentage similar to that of the wild-type mouse group (Fig. 4b). The Morris water maze was used to measure hippocampus-dependent spatial learning and memory among mouse groups. During the 5 days training period, 5xFAD mice showed less ability to learn the location of the platform compared to the learning ability of wild-type and DTMB-treated mice (Fig. 4c). To test spatial memory, the platform was removed, and target quadrant occupancy was measured on day 6. Although the target quadrant occupancy of transgenic mice was lower than that of wild-type mice, DTMB-treated transgenic mice showed markedly improved target quadrant occupancy, and thus memory function (Fig. 4d, e). These results demonstrate that DTMB improves spatial learning and memory in 5xFAD mouse model.

### DTMB Ameliorates Aβ Pathology in the 5xFAD Mouse Brain

**Fig. 5:**
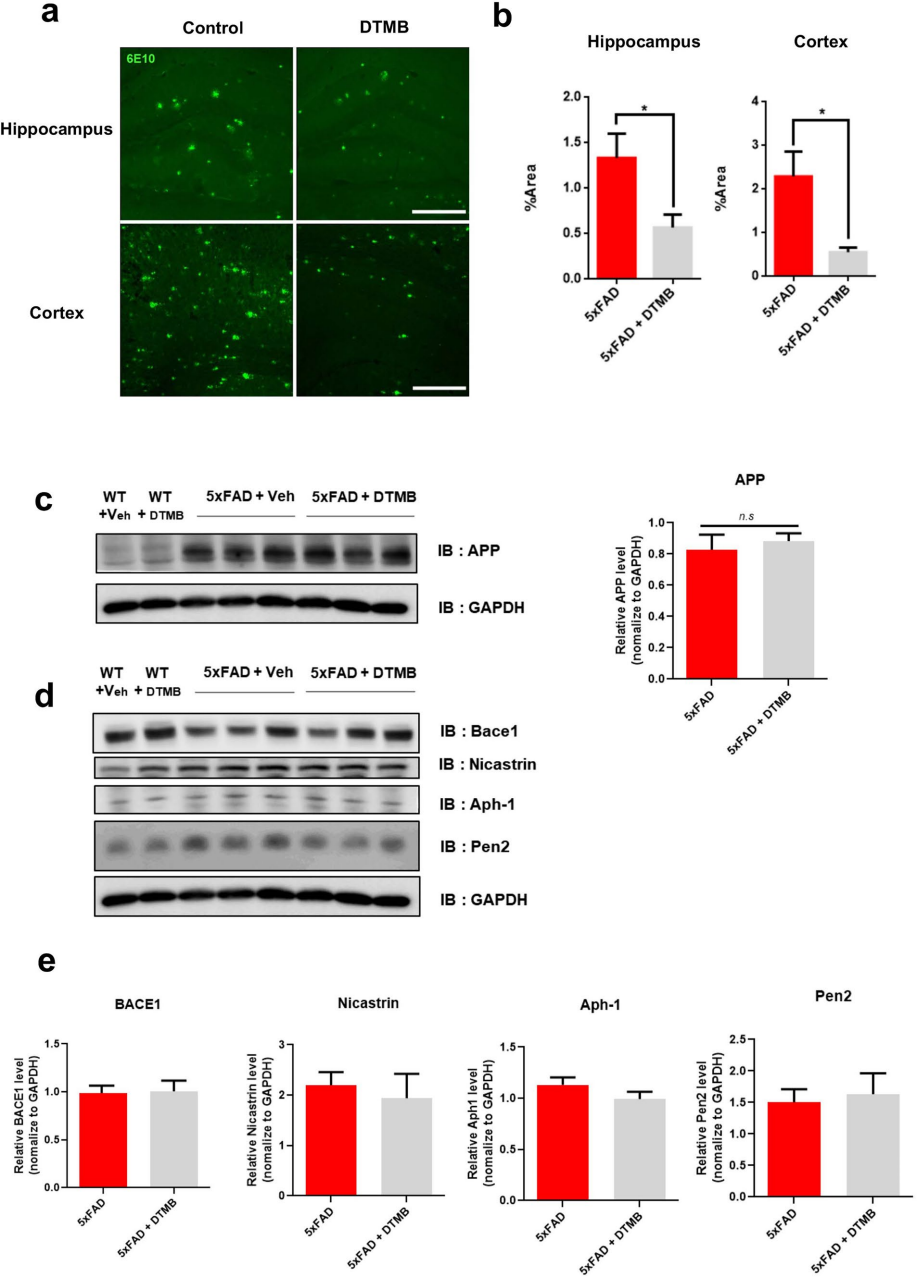
Aβ pathology in DTMB-treated 5xFAD mice. **a**, **b** Aβ plaques were detected using 6e10 antibody in both the hippocampal and the cortical tissue samples. Fluorescence (% area) was measured using ImageJ, scale bar = 200 µm. **c** Representative image of the Western blot is shown. Total APP level in the hippocampal tissue was measured through Western blotting to compare mice between the 5xFAD control group and DTMB-treated 5xFAD group. **d**, **e** Representative Western blot data to confirm the protein level of enzymes related to APP processing are shown (BACE1 for β-secretase, nicastrin1, aph-1, and pen2 for γ-secretase). Quantification was analyzed using ImageJ. All data were shown as the means ± standard error of the mean (SEM) **p < 0.01, ***p < 0.001 by *t* test

Aβ pathology is prominent hallmark of Alzheimer’s disease in both mouse model and patient’s brain [2, 3]. To examine whether DTMB decreases Aβ pathology, histological analyses were conducted using brain tissue obtained from 5xFAD mice. The amount of Aβ aggregates in the hippocampus and cortex of DTMB-treated mice was significantly decreased compared to that of transgenic control mice (Fig. 5a, b). The Aβ peptide is produced from amyloid precursor protein (APP) by the actions of β- and γ-secretase [2]. There lies a possibility in which DTMB acts on APP processing to inhibit the production of Aβ aggregates. To investigate this possibility, we measured the protein levels of total APP and components of both β- and γ-secretase in the cortex and hippocampus through western blot and qPCR. Interestingly, there were neither changes in APP protein and mRNA level nor changes in the protein levels of β- and γ-secretase (Fig. 5c, e and Supplementary data 4a–c) in the hippocampus. The protein level of β-secretase was slightly decreased in the cortex of DTMB-treated mice. In addition, amyloid beta monomer was not decreased by DTMB treatment, but Aβ aggregates in the insoluble fraction were decreased (Supplementary data 4d, e). These data indicate that DTMB reduces Aβ aggregates in the brain independent of the APP processing pathway.

### DTMB Reduces a Chronic Inflammatory Marker in 5xFAD Mouse Brain

**Fig. 6:**
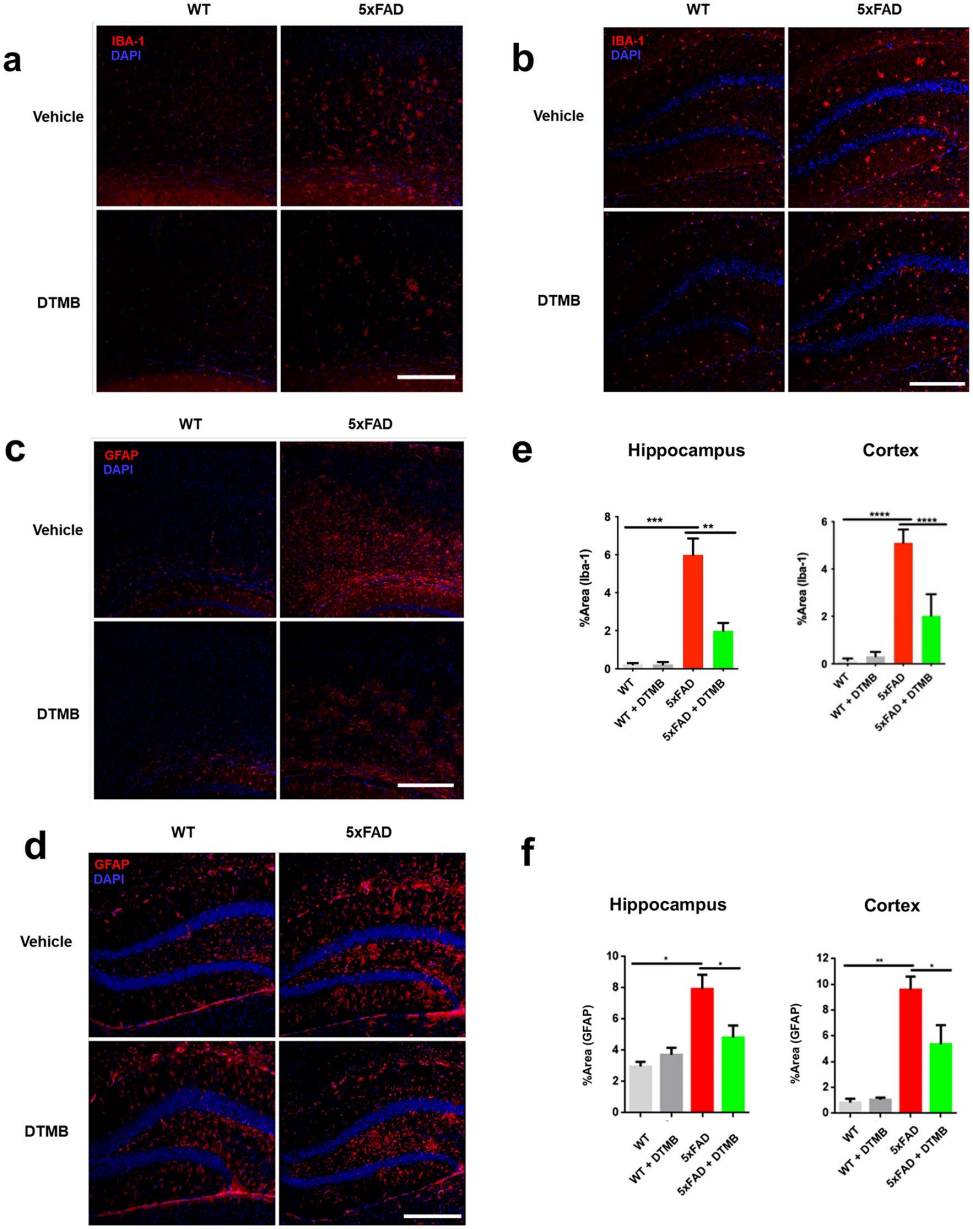

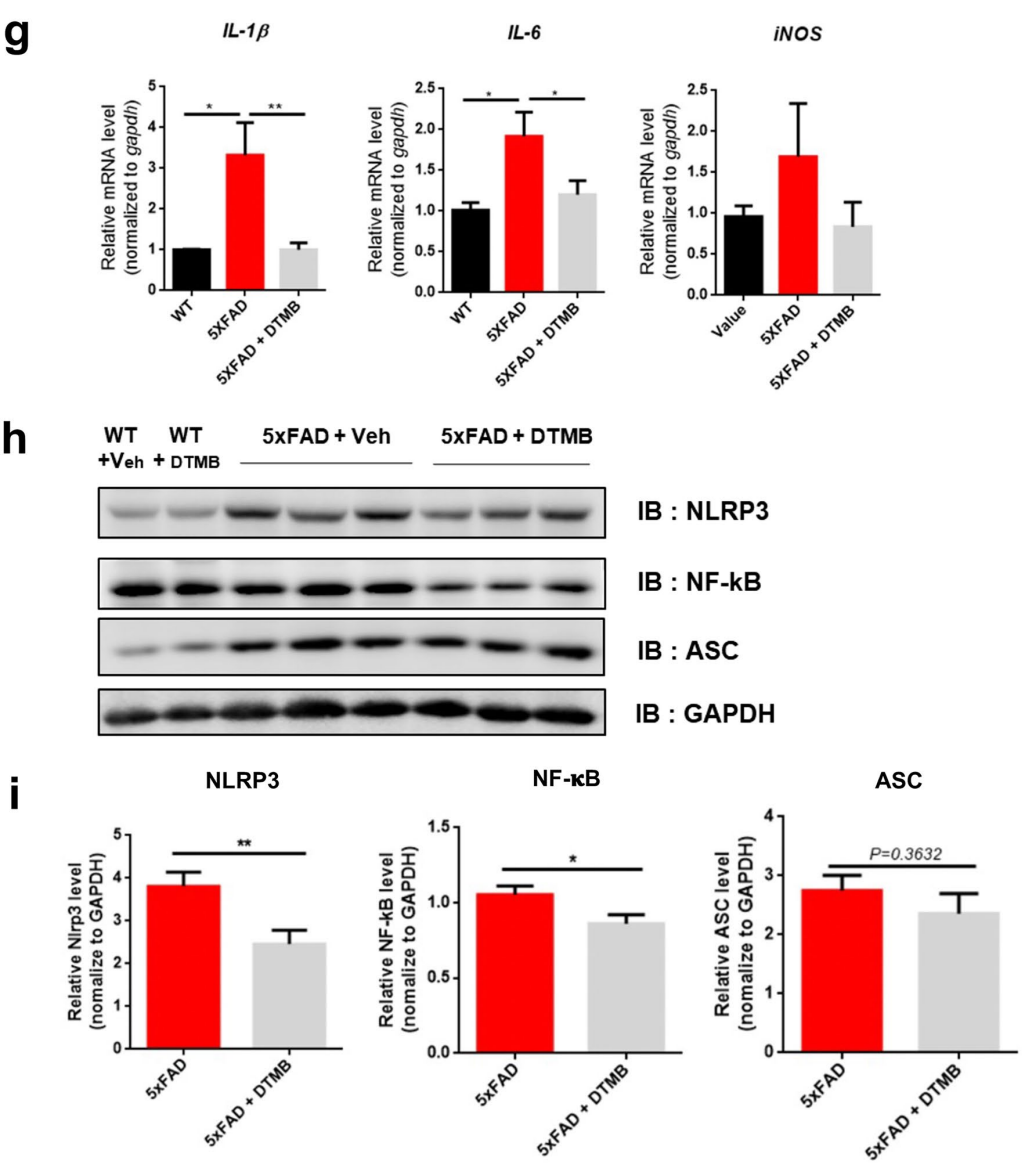
DTMB reduces chronic inflammation pathology in 5xFAD mouse brain. **a**, **b** Representative immunohistochemical images of Iba-1 staining in the cortex and hippocampus of 5xFAD mice treated with either vehicle or DTMB. Red fluorescence intensity represents the level of microglia gliosis. **c**, **d** Representative immunohistochemical images of GFAP staining in the cortex and hippocampus of 5xFAD mice treated with either vehicle or DTMB. Red fluorescent intensity shows the level of astrocyte gliosis. **e**, **f** Quantification graph of the immunohistochemical images showing the percentage area of fluorescence analyzed by Image. **g** Quantitative PCR data of proinflammatory cytokines and related enzyme. **h**, **i** Protein levels of inflammasome-related proteins (NLRP3, NF-κB, and ASC) in hippocampal brain lysate by western blot. All data are shown as mean ± standard error of the mean (SEM) **p < 0.01, ***p < 0.001 by one-way ANOVA and *t* test

As we have shown in previous experiments that the reduction of Aβ plaques by DTMB was not due to the regulation of the APP processing mechanism, we next looked into other possible modes of action. Recently, relationship between Aβ pathology and chronic inflammation has demonstrated in many studies. Chronic inflammation contributes to the production of proinflammatory cytokines overactivating both microglia and astrocyte that then lose the ability to remove Aβ aggregates under the pathological condition [35]. In addition, inflammasome components can accelerate the progress of Aβ pathology by being a seed of Aβ aggregation. Therefore, we confirmed whether the formation of Aβ plaques was reduced by DTMB-mediated suppression of neuroinflammation as DTMB exerts an anti-inflammatory effect on microglia (Fig. 3). We first observed the effect of DTMB on the gliosis of microglia and astrocyte in the brain tissue of 5xFAD model. During inflammatory conditions, the number of activated microglia and astrocyte is markedly increased in the brain of patients with Alzheimer’s disease and 5xFAD mouse model [36, 37]. We confirmed the level of gliosis for microglia and astrocyte by measurement fluorescence intensity of Iba-1 and GFAP, and found that the percentage of areas stained was decreased in DTMB-treated transgenic mice compared to transgenic controls (Fig. 6a–f). In addition, the expression levels of proinflammatory cytokines, and their related enzymes (*Il-1β*, *Il-6*, and *Inos*), were also decreased in DTMB-treated transgenic mice (Fig. 6g). Similar results were obtained from primary microglia (Fig. 3). In hippocampal brain lysates, the protein levels of NLRP3 and NF-κB were decreased, but the level of ASC was not changed significantly (Fig. 6h, i). Those results suggest that DTMB decreases chronic inflammation pathology in a mouse model of Alzheimer’s disease.

### DTMB Changes Global Genetic Expression Related to Chronic Inflammation and Synaptic Function

**Fig. 7:**
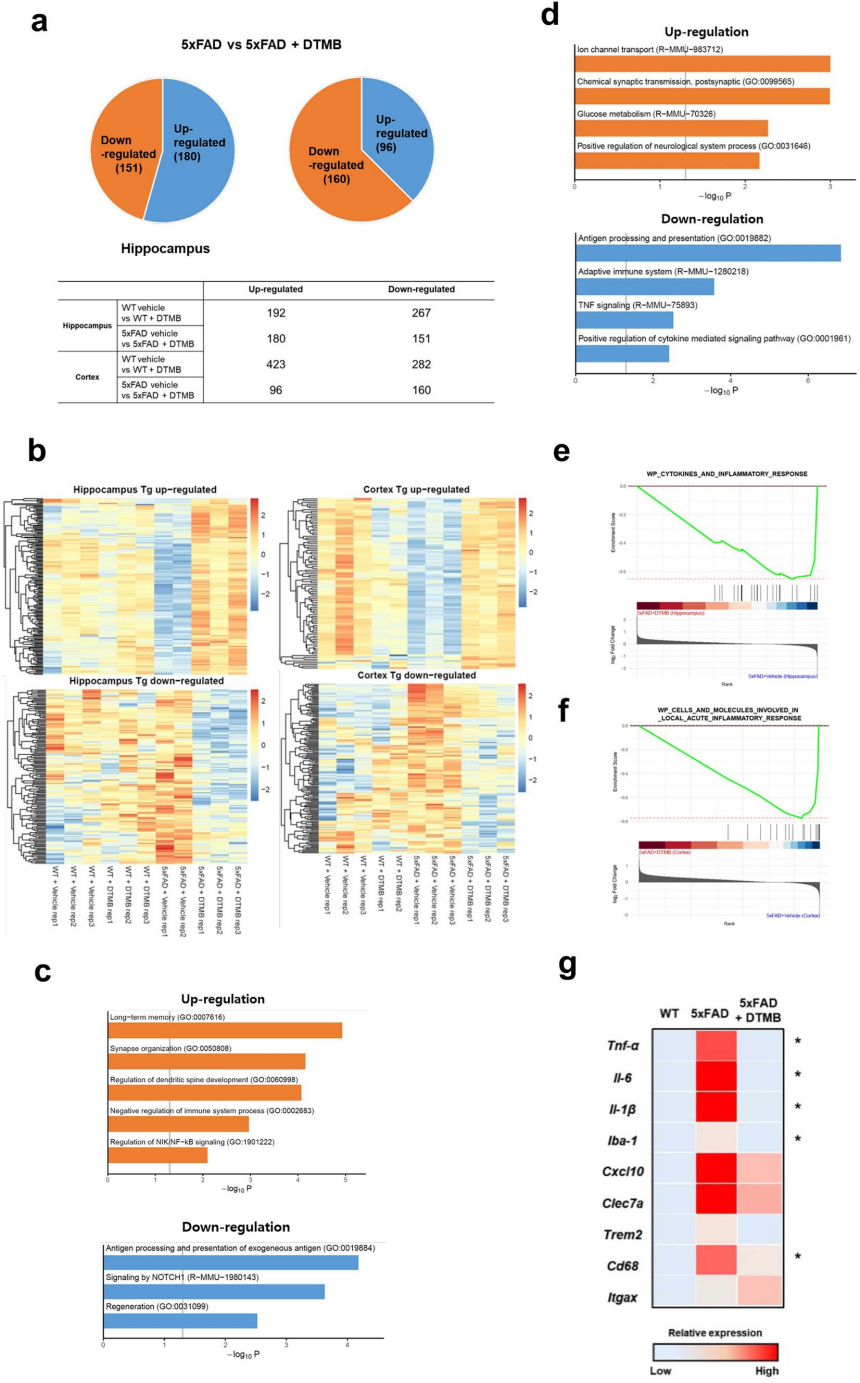
Bulk RNA-seq data of DTMB-treated 5xFAD mouse brain. **a** Overview of differentially expressed genes (DEGs). **b** Heat map of DEGs from hippocampus and cortex. **c**, **d** Gene ontology (GO) analysis. The top enriched GO terms of either upregulated or downregulated genes were analyzed from hippocampus and cortex of DTMB- or vehicle-treated 5xFAD mice. **e**, **f** GSEA enrichment plot (Wiki pathway). Inflammatory response-related gene sets were more enriched in the hippocampus and cortex of mice from the 5xFAD group treated with vehicle than that found in mice from the DTMB-treated 5xFAD group. Hippocampus analysis: NES score =  − 2.44, *p* < 0.001, and FDR < 0.001. Cortex brain analysis: NES score =  − 2.57, *p* < 0.001, and FDR < 0.001. **g** Heat map of gene expression related to microglial activation in adult microglia. Adult microglia were sorted from vehicle- or DTMB-treated 5xFAD mice using MACs system. Quantitative PCR was performed to analyze the gene expression related to chronic inflammation and microglial activation. Data represent the mean ± standard error of the mean (SEM) of three independent experiments. *p < 0.05; **p < 0.01; ***p < 0.001, by one-way ANOVA

In the previous results, we determined that DTMB has an anti-inflammatory effect on microglia and improves the memory deficit exhibited in 5xFAD mice. To gain insights on global gene expression in the brain tissue, bulk RNA-seq was conducted by extracting whole RNA from the cortex and hippocampus of mice from four mouse groups (WT, WT + DTMB, TG, and TG + DTMB). The gene expression cluster for each group showed a high correlation in the principal component analysis (PCA) plot. The gene expression pattern of DTMB-treated 5xFAD mice was similar to that of wild-type mice (Supplementary data 5a). A comparison of gene expression changes between transgenic and DTMB-treated transgenic mice shows that 180 genes were upregulated and 151 genes were downregulated in hippocampus (Fig. 7a and Supplementary data 5a). In cortex samples, 96 genes were upregulated and 160 genes were downregulated (Fig. 7a). We performed GO analysis to confirm the function of differentially expressed genes and found that upregulated genes were mainly identified in the functional categories of long-term memory and synapse function, and that genes upregulated in the hippocampus and cortex were related to the negative regulation of the immune system including NF-κB signaling (Fig. 7b–d). By contrast, genes related to chronic inflammation, like antigen processing and the TNF-α signaling pathway, were markedly downregulated in the cortex of DTMB-treated mice. Representative enrichment plots also showed that inflammation-related genes are enriched in 5xFAD transgenic mice (Fig. 7e, f). To assess the gene expression related to inflammation and microglial activation, we sorted adult microglia from wild-type, 5xFAD, or DTMB-treated 5xFAD mice using CD11b magnetic bead and performed RT-qPCR. In line with results of BV2 cells and primary microglia (Fig. 3b–e and Supplementary data 2b), RT-qPCR analysis showed that the levels of proinflammatory cytokines (*Tnf-α*, *Il-6*, and *Il-1β*) and microglial activation markers (*Iba-1*, *Trem2*, and *Cd68*) were markedly decreased in microglia from DTMB-treated 5xFAD mice (Fig. 7g). Further, analysis of our list of differentially expressed gene revealed that target genes of all PPAR subtypes were induced in hippocampus and cortex (Supplementary data 5d). In addition, the enrichment scores of gene expression related to the PPAR signaling pathway and glucose-lipid metabolism were enhanced in DTMB-treated mice (Supplementary data 5e). These data support that DTMB decreases gene expression related with chronic inflammation, but increases gene expression involved in synaptic function as a pan-PPAR agonist in the brain of 5xFAD mice.

## Discussion

In this study, the novel synthetic pan-PPAR ligand DTMB was identified and its potential as a therapeutic agent for Alzheimer’s disease was further demonstrated. DTMB decreased the production of proinflammatory cytokines in the microglia and microglial activation in the brain tissue of DTMB-treated mice. In addition, orally administrated DTMB improved cognitive functions and Aβ pathology in the 5xFAD mice, without altering the APP processing. The mechanism behind such results elicited by DTMB could be explained by an anti-inflammatory effect of DTMB. Chronic neuroinflammation is considered one of the main reasons of the pathological progression of Alzheimer’s diseases [11]. Heneka et al. have reported that neuroinflammation can accelerate Aβ aggregation and microglia toxicity in the pathological condition of Alzheimer’s disease since increased inflammasome components could act as seeds for Aβ aggregation [35, 38]. In addition, activated NLRP3 and excessive cytokine production by chronic neuroinflammation incrementally leads to overactivated microglia, which eventually lose their function in reducing the Aβ aggregates [39, 40]. As shown in Fig. 7g, while the microglial activation markers (*Iba-1*, *cxcl10*, *clec7a*, and *trem2*) and proinflammatory cytokines were increased in the microglia of 5xFAD mice, they were decreased in the microglia of DTMB-treated 5xFAD mice. Based on these facts, it could be expected that the anti-inflammatory effect of DTMB on microglia has reduced the amyloid beta pathology.

Another possible mechanism behind the reduction of Aβ pathology by DTMB is the regulation of Aβ degrading enzyme. Previously, it has been reported that selective PPARγ agonist like losartan can decrease Aβ pathology by enhancing the Aβ degrading enzymes, like neprilysin and insulin-degrading enzyme (IDE) [41, 42]. Since DTMB also acts as a partial PPARγ agonist, we checked the differentially expressed genes through RNAseq experiment to confirm whether Aβ pathology is reduced by downregulation of amyloid beta degrading enzymes (Supplementary data 6). When we compared the DEG list from hippocampus and cortex of DTMB- or vehicle-treated 5xFAD mice, the expression of *neprilysin* was increased in the hippocampus and cortex tissues of DTMB-treated 5xFAD mice, although the change was not statistically significant (*p*-value is 0.27). In addition, gene expression level of *Ece1* was significantly increased in the hippocampus tissue of DTMB-treated mice. Based on these results from RNAseq, DTMB could potentially decrease Aβ pathology by enhancing Aβ clearance enzyme, although it may not be considered as the major mechanism of DTMB action.

Peripheral inflammation also leads to cognitive dysfunction as the proinflammatory cytokines and immune cells can penetrate from circulating blood into the brain. Metabolic dysfunction including impairment of glucose metabolism and insulin resistance is a main cause of peripheral inflammation [43]. By inducing insulin resistance using a high-fat diet in an Alzheimer’s disease mouse model (APP/PS1), neuroinflammation and Aβ pathology were more severe compared to mice from the normal diet group [44]. Patients with type 2 diabetes mellitus also have a high risk of developing Alzheimer’s disease and share a similar biological pathology [45]. Previously, PPAR has been considered a drug target to treat metabolic disorders, particularly the type 2 diabetes. As shown in DTMB-treated mouse brain, DTMB agonistically binds to all PPAR subtypes (Fig. 1) and can induce gene expression involved in glucose and lipid metabolism related to PPAR signaling (Supplementary data 5D, E). In fact, although DTMB may have a higher binding affinity towards PPARα (Fig. 1), the target genes of PPAR subtypes were upregulated in the cortex and hippocampus of DTMB-treated mice (Supplementary data 5E). These results indicate that DTMB can act as a pan-agonist of PPAR in an in vivo animal model. Therefore, it is possible that DTMB alleviates the pathological condition of Alzheimer’s disease by reducing the peripheral inflammation caused by metabolic dysfunction through PPAR signaling.

Recently, many studies reported that nitric oxide stress through S-nitrosylation on target proteins contributes to synaptic loss and neuronal cell death in Alzheimer’s disease [46–49]. Excessive nitric oxide is one of the final products of chronic inflammation, which leads to neuronal cell death and synaptic failure in the central nervous system [48]. Nitric oxide can be transferred to cysteine and tyrosine residue of Drp1, which is a mitochondrial fission enzyme, and accelerates excessive fragmentation of mitochondria resulting in synaptic loss [49]. We observed that DTMB decreased the production of nitrite and gene expression of iNOS by inhibiting NF-κB signaling pathway. These results support that DTMB can attenuate synaptic loss by inhibiting nitrosative stress in the brain of Alzheimer’s disease model mice.

All subtypes of PPAR could be a therapeutic target for Alzheimer’s disease and some of their agonists are in the clinical study being developed as therapeutic agents [50]. Fibrate family is a family of selective PPARα agonists that include WY-14643, fenofibrate, and pemafibrate. It has been reported that fibrate compounds have anti-inflammatory effect on microglia and improve cognitive functions [51, 52]. Gemfibrozil is also a PPARα activating fibrate that had an effect in improving the memory dysfunction in 5xFAD mice model [53], but failed in phase I clinical trial. PPAR-β/δ agonists are also part of drug development for Alzheimer’s disease. GW0742, which is selective PPAR-β/δ agonist, decreases astrocyte activation and Aβ pathology [54]. T3D-959 is a dual agonist of PPAR-β/δ and PPARγ with 15-fold higher selectivity to PPAR-β/δ that decreases Aβ pathology and neuroinflammation [55]. According to Alzheimer’s disease drug development pipeline reports in year 2022, T3D-959 is under the phase II clinical trials as therapeutic agents for Alzheimer’s disease [56]. Furthermore, telmisartan and losartan are selective PPARγ agonists that are currently under phase 1 and 3 clinical trials, respectively [56]. Especially, losartan decreases Aβ pathology and neuroinflammation in APP/PS1 mice model [42, 57] as an attenuating agent of angiotensin receptor signaling. Inhibition of angiotensin receptor II signaling improves the neuroinflammation by regulating microglial TLR4 and NF-κB signaling in the brain [58, 59]. However, the underlying mechanism of how losartan regulates angiotensin receptor signaling through PPARγ is not clear. In view of these trials, PPAR is also considered a good candidate as a therapeutic solution for Alzheimer’s disease.

Neuroinflammation is also related with the onset of several other neurodegenerative disorders like Parkinson’s disease [60], amyotrophic lateral sclerosis [61], and multiple sclerosis [62]. For further study, experiments testing the toxicity and safety of DTMB should be conducted and evaluated as DTMB is a promising candidate for neurodegenerative disease.

## Supplementary Information

Below is the link to the electronic supplementary material.

Supplementary file1 (DOCX 29 kb)

Supplementary file2 (PDF 508 kb)

## References

[1] McKhann GM, Knopman DS, Chertkow H (2011). The diagnosis of dementia due to Alzheimer’s disease: recommendations from the National Institute on Aging-Alzheimer’s Association workgroups on diagnostic guidelines for Alzheimer’s disease. Alzheimers Dement.

[2] Long JM, Holtzman DM (2019). Alzheimer disease: an update on pathobiology and treatment strategies. Cell.

[3] Congdon EE, Sigurdsson EM (2018). Tau-targeting therapies for Alzheimer disease. Nat Rev Neurol.

[4] Umeda T, Ramser EM, Yamashita M (2015). Intracellular amyloid β oligomers impair organelle transport and induce dendritic spine loss in primary neurons. Acta Neuropathol Commun.

[5] Franco R, Cedazo-Minguez A (2014). Successful therapies for Alzheimer’s disease: why so many in animal models and none in humans?. Front Pharmacol.

[6] Cummings J, Lee G, Ritter A, Sabbagh M, Zhong K (2020). Alzheimer’s disease drug development pipeline: 2020. Alzheimer’s Dement: Transl Res Clin Interv.

[7] Furman D, Campisi J, Verdin E (2019). Chronic inflammation in the etiology of disease across the life span. Nat Med.

[8] Chen L, Deng H, Cui H (2018). Inflammatory responses and inflammation-associated diseases in organs. Oncotarget.

[9] Demine S, Schiavo AA, Marín-Cañas S, Marchetti P, Cnop M, Eizirik DL (2020). Pro-inflammatory cytokines induce cell death, inflammatory responses, and endoplasmic reticulum stress in human iPSC-derived beta cells. Stem Cell Res Ther.

[10] McCoy MK, Tansey MG (2008). TNF signaling inhibition in the CNS: implications for normal brain function and neurodegenerative disease. J Neuroinflammation.

[11] Schwartz M, Deczkowska A (2016). Neurological disease as a failure of brain–immune crosstalk: the multiple faces of neuroinflammation. Trends Immunol.

[12] Galloway DA, Phillips AE, Owen DR, Moore CS (2019). Phagocytosis in the brain: homeostasis and disease. Front Immunol.

[13] Perry VH, Holmes C (2014). Microglial priming in neurodegenerative disease. Nat Rev Neurol.

[14] Butovsky O, Weiner HL (2018). Microglial signatures and their role in health and disease. Nat Rev Neurosci.

[15] Tyagi S, Gupta P, Saini AS, Kaushal C, Sharma S (2011). The peroxisome proliferator-activated receptor: a family of nuclear receptors role in various diseases. J Adv Pharm Technol Res.

[16] Staels B, Fruchart J-C (2005). Therapeutic roles of peroxisome proliferator–activated receptor agonists. Diabetes.

[17] Clark RB (2002). The role of PPARs in inflammation and immunity. J Leukoc Biol.

[18] Delerive P, De Bosscher K, Besnard S (1999). Peroxisome proliferator-activated receptor α negatively regulates the vascular inflammatory gene response by negative cross-talk with transcription factors NF-κB and AP-1. J Biol Chem.

[19] Zingarelli B, Piraino G, Hake PW (2010). Peroxisome proliferator-activated receptor δ regulates inflammation via NF-κB signaling in polymicrobial sepsis. Am J Pathol.

[20] Wang K, Li Y-F, Lv Q, Li X-M, Dai Y, Wei Z-F (2018). Bergenin, acting as an agonist of PPARγ, ameliorates experimental colitis in mice through improving expression of SIRT1, and therefore inhibiting NF-κB-mediated macrophage activation. Front Pharmacol.

[21] Hou Y, Moreau F, Chadee K (2012). PPARγ is an E3 ligase that induces the degradation of NFκB/p65. Nat Commun.

[22] Dallakyan S, Olson AJ. Small-molecule library screening by docking with PyRx. In: Chemical biology: methods and protocols*.* Hempel JE, Williams CH, Hong CC (Eds.) (Springer New York, New York, NY, 2015) 243–250. 10.1007/978-1-4939-2269-7_19 25618350

[23] Trott O, Olson AJ (2010). AutoDock Vina: improving the speed and accuracy of docking with a new scoring function, efficient optimization, and multithreading. J Comput Chem.

[24] Lian H, Roy E, Zheng H. Protocol for primary microglial culture preparation. Bio-protocol. 2016;6(21). 10.21769/BioProtoc.1989 PMC5669279 29104890

[25] Martin M (2011). Cutadapt removes adapter sequences from high-throughput sequencing reads. EMBnet journal.

[26] Dobin A, Davis CA, Schlesinger F (2013). STAR: ultrafast universal RNA-seq aligner. Bioinformatics.

[27] Li B, Dewey CN (2011). RSEM: accurate transcript quantification from RNA-seq data with or without a reference genome. BMC Bioinform.

[28] Love MI, Huber W, Anders S (2014). Moderated estimation of fold change and dispersion for RNA-seq data with DESeq2. Genome Biol.

[29] Zhou Y, Zhou B, Pache L (2019). Metascape provides a biologist-oriented resource for the analysis of systems-level datasets. Nat Commun.

[30] Subramanian A, Tamayo P, Mootha VK (2005). Gene set enrichment analysis: a knowledge-based approach for interpreting genome-wide expression profiles. Proc Natl Acad Sci.

[31] Liu T, Zhang L, Joo D, Sun S-C (2017). NF-κB signaling in inflammation. Signal Transduct Target Ther.

[32] Broz P, Dixit VM (2016). Inflammasomes: mechanism of assembly, regulation and signalling. Nat Rev Immunol.

[33] Spangenberg E, Severson PL, Hohsfield LA (2019). Sustained microglial depletion with CSF1R inhibitor impairs parenchymal plaque development in an Alzheimer’s disease model. Nat Commun.

[34] Davis K (2002). NSAID and Alzheimer’s disease; possible answers and new questions. Mol Psychiatry.

[35] Venegas C, Kumar S, Franklin BS (2017). Microglia-derived ASC specks cross-seed amyloid-β in Alzheimer’s disease. Nature.

[36] Heneka MT, Carson MJ, El Khoury J (2015). Neuroinflammation in Alzheimer’s disease. Lancet Neurol.

[37] Wyss-Coray T (2006). Inflammation in Alzheimer disease: driving force, bystander or beneficial response?. Nat Med.

[38] Friker LL, Scheiblich H, Hochheiser IV (2020). β-Amyloid clustering around ASC fibrils boosts its toxicity in microglia. Cell Rep.

[39] Minter MR, Taylor JM, Crack PJ (2016). The contribution of neuroinflammation to amyloid toxicity in Alzheimer’s disease. J Neurochem.

[40] Heneka MT, Kummer MP, Stutz A (2013). NLRP3 is activated in Alzheimer’s disease and contributes to pathology in APP/PS1 mice. Nature.

[41] Drews HJ, Yenkoyan K, Lourhmati A (2019). Intranasal losartan decreases perivascular beta amyloid, inflammation, and the decline of neurogenesis in hypertensive rats. Neurotherapeutics.

[42] Drews HJ, Klein R, Lourhmati A, et al. Losartan improves memory, neurogenesis and cell motility in transgenic Alzheimer’s mice. Pharmaceuticals (Basel). 2021;14(2). 10.3390/ph14020166 PMC7923419 33672482

[43] Cai D, Liu T (2012). Inflammatory cause of metabolic syndrome via brain stress and NF-κB. Aging (Albany NY).

[44] Kim D-G, Krenz A, Toussaint LE (2016). Non-alcoholic fatty liver disease induces signs of Alzheimer’s disease (AD) in wild-type mice and accelerates pathological signs of AD in an AD model. J Neuroinflammation.

[45] Barbagallo M, Dominguez LJ (2014). Type 2 diabetes mellitus and Alzheimer’s disease. World J Diabetes.

[46] Nakamura T, Tu S, Akhtar MW, Sunico CR, Okamoto S-I, Lipton SA (2013). Aberrant protein s-nitrosylation in neurodegenerative diseases. Neuron.

[47] Seneviratne U, Nott A, Bhat VB (2016). S-nitrosation of proteins relevant to Alzheimer’s disease during early stages of neurodegeneration. Proc Natl Acad Sci.

[48] Lipton SA, Choi Y-B, Pan Z-H (1993). A redox-based mechanism for the neuroprotective and neurodestructive effects of nitric oxide and related nitroso-compounds. Nature.

[49] Nakamura T, Oh C-K, Liao L, et al. Noncanonical transnitrosylation network contributes to synapse loss in Alzheimer’s disease. Science. 2021;371(6526). 10.1126/science.aaw0843 PMC8091809 33273062

[50] Bright JJ, Kanakasabai S, Chearwae W, Chakraborty S (2008). PPAR regulation of inflammatory signaling in CNS diseases. PPAR Res.

[51] Esmaeili MA, Yadav S, Gupta RK (2016). Preferential PPAR-α activation reduces neuroinflammation, and blocks neurodegeneration in vivo. Hum Mol Genet.

[52] Ogawa K, Yagi T, Guo T (2020). Pemafibrate, a selective PPARα modulator, and fenofibrate suppress microglial activation through distinct PPARα and SIRT1-dependent pathways. Biochem Biophys Res Commun.

[53] Chandra S, Pahan K (2019). Gemfibrozil, a lipid-lowering drug, lowers amyloid plaque pathology and enhances memory in a mouse model of Alzheimer’s disease via peroxisome proliferator-activated receptor α. J Alzheimers Dis Rep.

[54] Konttinen H, Gureviciene I, Oksanen M (2019). PPARβ/δ-agonist GW0742 ameliorates dysfunction in fatty acid oxidation in PSEN1ΔE9 astrocytes. Glia.

[55] de la Monte SM, Tong M, Schiano I, Didsbury J (2017). Improved brain insulin/IGF signaling and reduced neuroinflammation with T3D–959 in an experimental model of sporadic Alzheimer’s disease. J Alzheimers Dis.

[56] Cummings J, Lee G, Nahed P (2022). Alzheimer’s disease drug development pipeline: 2022. Alzheimer’s Dement: Transl Res Clin Interv.

[57] Salmani H, Hosseini M, Beheshti F (2018). Angiotensin receptor blocker, losartan ameliorates neuroinflammation and behavioral consequences of lipopolysaccharide injection. Life Sci.

[58] Biancardi VC, Stranahan AM, Krause EG, de Kloet AD, Stern JE (2016). Cross talk between AT1 receptors and Toll-like receptor 4 in microglia contributes to angiotensin II-derived ROS production in the hypothalamic paraventricular nucleus. Am J Physiol Heart Circ Physiol.

[59] Benicky J, Sánchez-Lemus E, Honda M (2011). Angiotensin II AT1 receptor blockade ameliorates brain inflammation. Neuropsychopharmacology.

[60] Rocha NP, de Miranda AS, Teixeira AL (2015). Insights into neuroinflammation in Parkinson’s disease: from biomarkers to anti-inflammatory based therapies. Biomed Res Int.

[61] Liu J, Wang F (2017). Role of neuroinflammation in amyotrophic lateral sclerosis: cellular mechanisms and therapeutic implications. Front Immunol.

[62] Kamma E, Lasisi W, Libner C, Ng HS, Plemel JR (2022). Central nervous system macrophages in progressive multiple sclerosis: relationship to neurodegeneration and therapeutics. J Neuroinflammation.

