# Supplementary material for: Synthetic PPAR Agonist DTMB Alleviates Alzheimer’s Disease Pathology by Inhibition of Chronic Microglial Inflammation in 5xFAD Mice
Source: Neurotherapeutics. 2022 Aug 2;19(5):1546–65. doi: 10.1007/s13311-022-01275-y (PMC9606171; doi:10.1007/s13311-022-01275-y)
Supplement: Supplementary file 1 — Supplementary file1 (DOCX 29 kb) [file 13311_2022_1275_MOESM1_ESM.docx]

**Supplementary data 1. A calibration standard curves and typical chromatograms of DTMB in the plasma and the brain.**

(a) Representative calibration curve of DTMB spiked in the mouse blank plasma in the concentration of 0.5 – 500 ng/mL. (b) Representative calibration curve of DTMB spiked in the mouse blank brain homogenate in the concentration of 0.5 – 100 ng/mL. r^2^ represents the correlation coefficient and p represents the statistical significance for the regression analysis. (c, d) Representative chromatograms of DTMB and berberine (IS) in mouse plasma and in brain homogenate of double blank, zero blank, standard calibration sample (0.5 ng/mL), and plasma or brain sample at 0.5 h following oral administration of DTMB (50 mg/kg).

**Supplementary data 2. Anti-inflammatory effect of DTMB.**

(a) MTT assay data. The HMO6, BV2, and RAW 264.7 cell lines were used to test DTMB cytotoxicity in a concentration-dependent manner. (b) Quantitative PCR analysis of mRNA expression of proinflammatory cytokines (*Il-6* and *Il-1β*), related enzyme (*Inos*), and M2-phenotype microglia marker (*Arg-1*). (c, d) Representative Western blot data of NF-κB, NLRP3, and ASC from primary microglia treated with either DMSO (0.1%) or DTMB (25 µM) for 24 h. LPS (1 µg/mL) was used to induce inflammatory responses. (e, f) Cells were treated with MG132 (20 µM) for 6 h to inhibit proteasomal degradation and treated with DTMB (25 µM) for 24 h. Quantification of blot intensity was measured using ImageJ. Data represents the mean ± standard error of the mean (SEM) of three independent experiments. *p < 0.05; **p < 0.01; ***p < 0.001, by one-way ANOVA

**Supplementary data 3. Changes in body weight and relative weight of organs after treatment of DTMB in 5xFAD mice model**

Effect of DTMB for 3 months on the body weight (a) and relative weights of tissues including brain (b), liver (c), heart (d), and spleen (e).

**Supplementary data 4. DTMB reduces Aβ pathology, but does not affect APP processing**

(a) Quantitative PCR analysis of mRNA expression of β-secretase (Bace-1) and γ-secretase (Aph1, Nicastrin, and Pen2). (b, c) Representative Western blot data of enzymes related to APP processing in a cortical lysate sample. Quantification was analyzed using ImageJ. All data are provided as means ± standard error of the mean (SEM) **p < 0.01, ***p < 0.001, by t test. (d) Representative Western blot data confirming the level of Aβ monomer using 6e10 antibody. (e) Representative dot blot data of the level of Aβ plaque in the insoluble fraction of brain lysates obtained from 5xFAD mice.

**Supplementary data 5. Bulk RNA-seq data of DTMB-treated 5xFAD mouse brain.** (a) Principal component analysis (PCA) plot. Gene expression patterns clearly formed similar clusters between samples of each mouse brain of the same group. (b, c) Gene ontology (GO) analysis. Top enriched GO terms of either upregulated or downregulated genes were analyzed from the hippocampus and cortex of DTMB- or vehicle-treated wild- type mice. (d) GSEA enrichment plot (KEGG database). Gene sets related to PPAR-related signaling as well as glucose and lipid metabolism were more enriched in the hippocampus and cortex of mice from 5xFAD group treated with DTMB compared with those found in vehicle-treated transgenic mice. PPAR signaling pathway gene term: NES score=1.04, p=0.433, and FDR=0.725. Fatty acid metabolism: NES score=1.33, p =0.0915, and FDR=0.255. Glycolysis and gluconeogenesis: NES score=1.18, p=0.201, and FDR=0.497. (e) Venn diagram of differentially expressed genes (DEGs) for PPAR target genes. Expression of PPAR target genes were upregulated in DTMB-treated brain.

**Supplementary data 6. DTMB has potential to decrease the A𝛃 degrading enzymes.**

The bar graphs show the average expression level of each gene detected by RNA-seq. The gene expression level of angiotensin-converting enzyme (*Ace*), endothelin converting enzymes-1 and -2 (*Ece-1* and *Ece-2*), insulin-degrading enzyme (*Ide*), and neprilysin (*Nep*) were compared to vehicle treated or DTMB treated mice brain (a) hippocampus and (b) cortex. Each dot represents the expression level of one replicate. The expression level is quantified as transcript per million (TPM) and significance of differential expression between DTMB- and vehicle-treated samples is represented as false discovery rate (FDR; Benjamini-Hochberg procedure).
